# A rare case of MGRS with immunotactoid glomerulopathy responding to bortezomib, dexamethasone, and rituximab

**DOI:** 10.1002/ccr3.3044

**Published:** 2020-06-22

**Authors:** Chaoyuan Kuang, Kevin Quann, Agnes K. Liman, Roy Frye, Nawras Alshoubaki, Mohan Ramkumar, Andrew D. Liman

**Affiliations:** ^1^ Hematology/Oncology section VA Pittsburgh Healthcare System Pittsburgh PA USA; ^2^ UPMC Hillman Cancer Center Pittsburgh PA USA; ^3^ Pathology and Laboratory Medicine VA Pittsburgh Healthcare System Pittsburgh PA USA; ^4^ Renal section VA Pittsburgh Healthcare System Pittsburgh PA USA

**Keywords:** immunotactoid glomerulopathy, monoclonal gammopathy of renal significance, nephrotic syndrome, Waldenstrom's macroglobulinemia

## Abstract

Patients with monoclonal gammopathy of renal significance should be treated with clone‐directed therapy against sources of monoclonal proteins in order to prevent progression to more advanced monoclonal gammopathies and renal failure.

## Graphical abstract

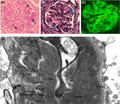

## 1 INTRODUCTION

Monoclonal gammopathy of renal significance (MGRS) is a recently described disorder caused by pathologic monoclonal protein (M‐protein) leading to renal disease but little hematologic disease burden.^1^ Failure to treat patients with MGRS can lead to poor outcomes due to progression of MGRS to more advanced monoclonal gammopathies, or end‐stage renal disease.^2^ We report a case of a 66‐year‐old woman with MGRS leading to nephrotic syndrome and immunotactoid glomerulopathy (ITG). We hypothesized that aggressive treatment of the patient's MGRS through B‐cell depletion and proteasome inhibition would improve her glomerulopathy and clinical outcomes. She was treated with bortezomib, dexamethaone, and rituximab with sustained normalization of proteinuria and circulating IgM levels.

## 2 CASE DESCRIPTION

**FIGURE 1:**
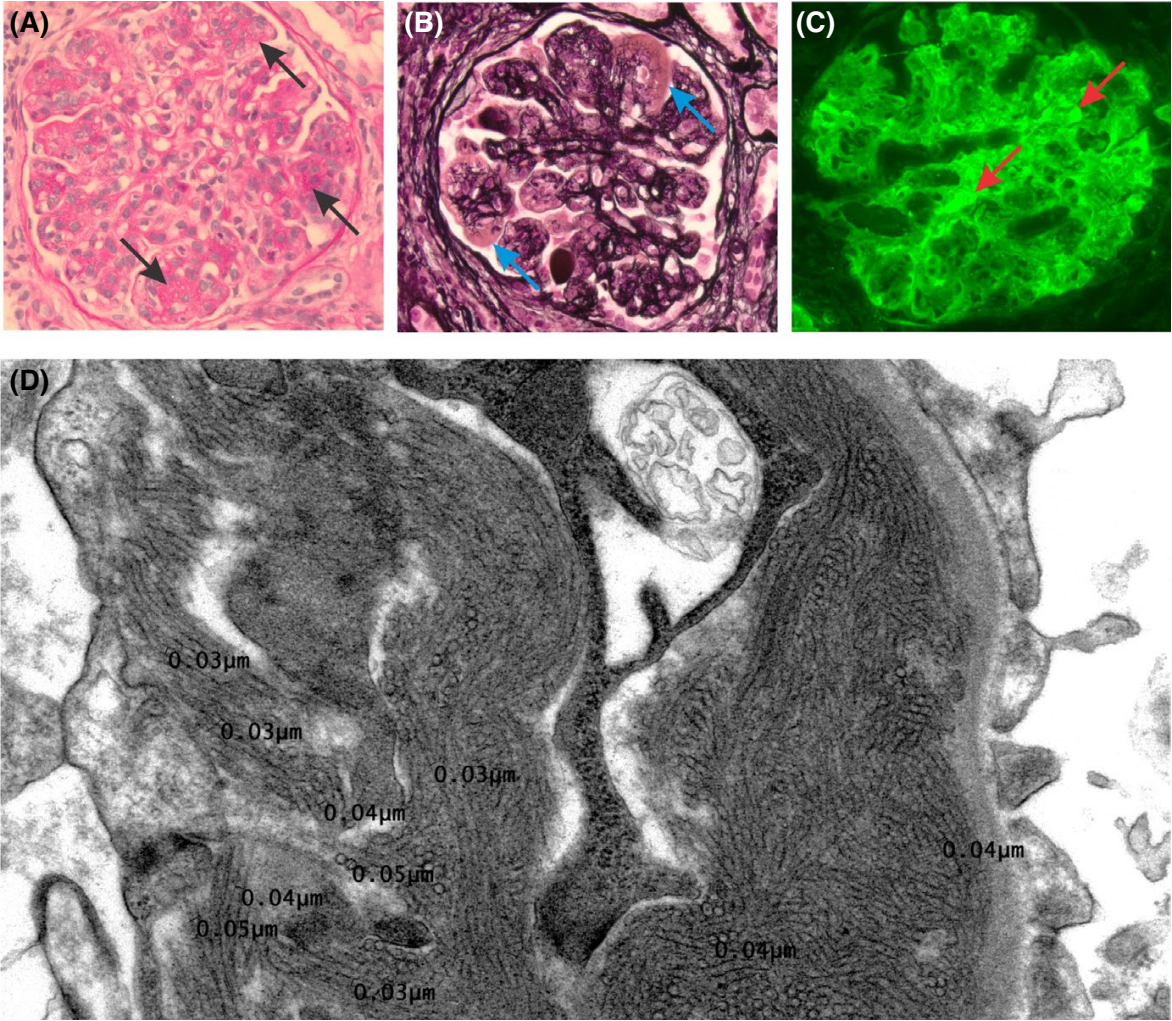
Microscopy of renal biopsy. A, PAS stain demonstrating mesangial expansion and mild segmental sclerosis (arrows). B, Jones stain demonstrating immune deposits (arrows) along glomerular capillaries which are negative for silver stain. C, Immunofluorescence stain for IgM showing 3+ staining on mesangial and capillary loops (arrows). D, Transmission electron microscopy showing numerous immunotactoid deposits composed of slightly curved, microtubular structures with mean diameter of 38.6 nm, hollow cores, and parallel alignment beneath the glomerular basement membrane and mesangium

A 66‐year‐old woman was referred to our hematology clinic in 2016 with chronic mild granulocytopenia dating back at least 5 years (absolute neutrophil count 1500‐2000 × 10^3^/µL) and new mild normocytic anemia (hemoglobin 11.1‐11.6 g/dL from baseline 12.0‐13.0 g/dL). Her history was notable for vitamin D deficiency and laparascopic hysterectomy, though she was otherwise healthy. Her workup including comprehensive metabolic panel, iron panel, vitamin B12, folate, fecal occult blood testing, and viral serologies was all unremarkable. However, serum protein electrophoresis identified a monoclonal IgM κ M‐protein at a concentration of 0.28 g/dL, free light chain ratio of 2.23 (normal 0.26‐1.65), with a serum‐free κ of 22.9 mg/L (normal 3.3‐19.4 mg/L). Quantitative immunoglobulins showed elevated IgM levels of 517 mg/dL (normal 43‐279 mg/dL) with low IgG (470 mg/dL, normal 791‐1643 mg/dL) and normal IgA (84 mg/dL, normal 66‐436 mg/dL). She was diagnosed with monoclonal gammopathy of uncertain significance and monitored. In 2018, two years after her initial hematology consultation, she presented to nephrology clinic with acute kidney injury (serum creatinine 1.39 mg/dL), peripheral edema, and hypertension. She had nephrotic range proteinuria at 6 g/24 hours and hematuria, though no Bence‐Jones proteins were detected. Her serum IgM level had climbed to 731 mg/dL at this time, with complement C3 low at 68 mg/dL (normal 79‐152 mg/dL). She underwent renal biopsy in April of 2018 which demonstrated immunotactoid glomerulopathy with membranoproliferative glomerulonephritis pattern (Figure 1A,B). Immunofluorescence of her renal biopsy showed κ light chain deposition in mesangial and capillary loops, with heavy IgM (Figure 1C) and moderate C3 staining (not shown). Electron microscopy revealed numerous immunotactoid deposits beneath the glomerular basement membrane (Figure 1D). Thioflavin and Congo Red staining were both negative for amyloid. Her serum M‐protein burden was unchanged. A bone marrow biopsy was obtained that was hypocellular (40%), though otherwise demonstrated normal trilineage hematopoiesis without immunohistochemical evidence of a monoclonal B‐cell population. However, flow cytometry performed on bone marrow aspirate did identify a small (<5%) CD20^+^, CD5^−^, CD10^−^, CD23^−^, and B‐cell population with κ light chain restriction. A CT of the neck, chest, abdomen, and pelvis was performed with IV contrast, and showed no pathological hepatosplenomegaly, lymphadenopathy, or lytic skeletal lesions to suggest Waldenstrom's Macroglobulinemia (WM) or myeloma.

**FIGURE 2:**
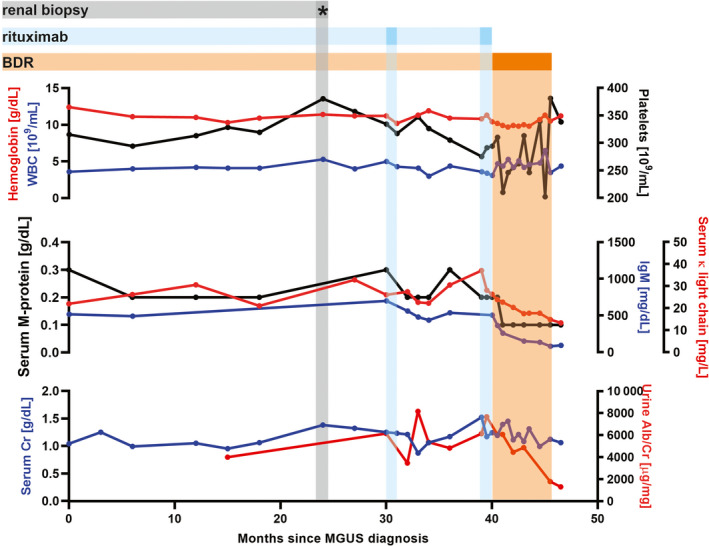
Treatment course and laboratory parameters. BDR, bortezomib + dexamethasone + rituximab

She was reclassified with MGRS and treated with rituximab 375 mg/m^2^ weekly, for four weeks, given over two separate courses. She experienced stable serum M‐protein and free light chain burden, improvement of creatinine clearance and hypertension. However, she continued to have nephrotic syndrome. During this initial treatment course, her urine albumin/creatinine ratio only showed mild improvement from 8166 mg/g in November 2018 to 7670 mg/g in June 2019. Due to her persistent renal dysfunction, we chose to treat with a WM‐inspired regimen consisting of bortezomib, dexamethasone, and rituximab, for a total of 5 cycles.^3^ Following treatment with this regimen, her serum IgM, serum‐free κ light chain, and creatinine have all normalized (92 mg/dL, 14.1 mg/L, and 1.06 mg/dL, respectively), while her urine albumin/creatinine ratio has improved dramatically to 1307 mg/g at time of this publication (Figure 2). Her serum M‐protein remains detectable at 0.1 g/dL, thereby meeting criteria for very good partial response per 6th International Workshop on WM.^4^

## 3 METHODS

This report is a retrospective chart review of one patient with MGRS and immunotactoid glomerulopathy. The patient reviewed this manuscript and provided written informed consent for the review of her medical records and publication of the results, per the Declaration of Helsinki.

Bortezomib, dexamethasone, and rituximab (BDR)^3^:

Cycle 1: bortezomib 1.3 mg/m^2^ IV on days 1, 4, 8, and 11; 21‐day cycle.

Cycles 2‐5: bortezomib 1.6 mg/m^2^ IV on days 1, 8, 15, and 22; 35‐day cycle.

Cycles 2 and 5 only: dexamethasone 40 mg PO on days 1, 8, 15, and 22; rituximab 375 mg/m^2^ IV on days 1,8, 15, and 22; 35‐day cycle.

Contact corresponding author for data acquisition and microscopy details.

## 4 RESULTS AND DISCUSSION

Monoclonal gammopathy of renal significance is a newer diagnostic classification encompassing patients who meet criteria for MGUS and otherwise have only renal disease caused by monoclonal gammopathy of a nonmalignant nature.^1^, ^2^ The spectrum of renal pathologies that fall under MGRS is evolving and is largely due to deposition of monoclonal Ig fragments in renal glomeruli.^5^ Immunotactoid glomerulopathy (ITG) is defined by the pathognomonic finding of organized microtubular deposits with diameters of 10‐60 nm,^5^, ^6^ as observed in our patient. ITG is frequently caused by IgG paraproteins and associated with CLL clones.^6^, ^7^ Our patient's workup revealed a CD5^−^, CD23^−^, and B‐cell clone, which precludes a CLL‐related premalignancy. Further, IgM κ light chain was found to be the unifying lesion in our patient's studies, making her case even more unusual. Although she was initially referred to hematology clinic for chronic mild granulocytopenia, it is unclear if this was in any way related to her underlying MGRS. The findings of mild anemia on her initial presentation may be best attributed to hematuria in the setting of a developing ITG.

Clinicians currently have no firm guidelines for treatment of MGRS or suspected MGRS. The hematologic outcomes of this disease entity were only recently described. A large retrospective analysis by Steiner and colleagues found decreased PFS and OS in pathologically proven MGRS patients as compared to MGUS patients.^2^ Despite an imbalanced cohort number due to the rareness of MGRS cases, the authors clearly demonstrated that MGRS patient have increased risk of progression to more advanced monoclonal gammopathies such as multiple myeloma or smoldering myeloma when looking across all Ig subtypes. However, it is known that patients with IgM MGUS are at higher risk for developing WM and non‐Hodgkin's lymphomas than their non‐IgM MGUS counterparts.^8^ Steiner and colleagues also found MGRS patients to have a higher risk of progression to end‐stage renal disease (ESRD) as compared to MGUS patients. This observation is in line with older data from Heilman and colleagues showing that 82% of MGUS with light chain deposition patients progressed to ESRD despite therapy.^9^ Thus, MGRS likely represents a distinct entity from MGUS with higher rates of both hematologic and renal disease progression.

While it is broadly understood that renal dysfunction in the setting of MGUS should be investigated with renal biopsy, under‐recognition of the increased risk of progression of MGRS as compared to MGUS can lead to undertreatment and even misclassification. Our patient presented with MGRS manifested as ITG, with classic signs of nephrotic syndrome. Typically, ITG is more closely associated with malignant conditions such as CLL, lymphoplasmacytic lymphoma, and multiple myeloma,^7^ and to our knowledge, there has only been one other case report of ITG associated with IgM MGRS. Gabbay and colleagues described a 83‐year‐old male patient who was initially diagnosed with MGUS and ITG 14 years prior to the report.^10^ He ultimately succumbed following progression of MGRS to WM and lymphoplasmacytic lymphoma. Given the evidence currently available in the literature, we assumed our patient to be at increased risk for progression to WM or ESRD and with her minimal burden of comorbidities; we determined that it was in her best interest to treat her disease aggressively to prevent these outcomes.

Very little prospective data are available to guide treatment for MGRS. Expert opinion currently recommends a clone‐directed approach.^1^, ^6^, ^11^ Gumber and colleagues reported in a recent case series over a dozen MGRS patients treated by this approach.^12^ Notably, nearly all of their patients had an IgG paraprotein or renal deposit identified, and most had no clonal involvement by bone marrow biopsy. Their treatment outcomes were encouraging, with most patients demonstrating at least partial response to rituximab‐based treatment regimens. We discussed upfront aggressive treatment with our patient and opted for rituximab monotherapy first in an attempt to avoid more toxic therapies. Due to the minimal renal response to rituximab, marked by persistent massive proteinuria, we proceeded with a bortezomib‐based regimen. We had also discussed employing a cyclophosphamide regimen given the patient's IgM paraprotein and renal dysfunction^11^; however, due to the patient's desire to avoid toxic therapies and take a step‐wise approach, we chose bortezomib, dexamethasone, and rituximab first.^3^ Currently available antiplasma cell and anti–B‐cell regimens would likely all have some activity against the broad spectrum of MGRS clones, and more studies are needed to define which regimens are best for each pathology.

In summary, we present a rare case of MGRS with ITG caused by IgM κ paraprotein from a non‐CLL B‐cell clone. Our patient has achieved a very good partial response to bortezomib, dexamethasone, and rituximab, and will be monitored for continued improvement. Monoclonal gammopathy of renal significance is an under‐recognized disease, but aggressive management may delay hematologic and renal progression of disease. To this end, more clinical studies are warranted to better define long‐term outcomes for these patients.

## CONFLICT OF INTEREST

None declared.

## AUTHOR CONTRIBUTIONS

CK and ADL: conceptualized this manuscript. CK: collected the data, and drafted and revised this manuscript. KQ: collected the data and revised this manuscript. ADL, AKL and RF: collected data and assisted in the writing. All authors were involved in revising this manuscript and approve of it in its final form.

## References

[1] Leung N , Bridoux F , Hutchison CA et al. Monoclonal gammopathy of renal significance: when MGUS is no longer undetermined or insignificant. Blood. 2012;120:4292‐4295. 23047823 10.1182/blood-2012-07-445304

[2] Steiner N , Göbel G , Suchecki P , Prokop W , Neuwirt H , Gunsilius E . Monoclonal gammopathy of renal significance (MGRS) increases the risk for progression to multiple myeloma: an observational study of 2935 MGUS patients. Oncotarget. 2018;9:2344‐2356. 29416776 10.18632/oncotarget.23412 PMC5788644

[3] Dimopoulos MA , García‐Sanz R , Gavriatopoulou M , et al. Primary therapy of Waldenström macroglobulinemia (WM) with weekly bortezomib, low‐dose dexamethasone, and rituximab (BDR): long‐term results of a phase 2 study of the European Myeloma Network (EMN). Blood. 2013;122:3276‐3282. 24004667 10.1182/blood-2013-05-503862

[4] Owen RG , Kyle RA , Stone MJ , et al. Response assessment in Waldenström macroglobulinaemia: update from the VIth International Workshop. Br J Haematol. 2013;160:171‐176. 23150997 10.1111/bjh.12102

[5] Bridoux F , Leung N , Hutchison CA , et al. Diagnosis of monoclonal gammopathy of renal significance. Kidney Int. 2015;87:698‐711. 25607108 10.1038/ki.2014.408

[6] Fermand J‐P , Bridoux F , Kyle RA , et al. How I treat monoclonal gammopathy of renal significance (MGRS). Blood. 2013;122:3583‐3590. 24108460 10.1182/blood-2013-05-495929

[7] Nasr SH , Fidler ME , Cornell LD , et al. Immunotactoid glomerulopathy: clinicopathologic and proteomic study. Nephrol Dial Transplant. 2012;27:4137‐4146. 22872726 10.1093/ndt/gfs348

[8] Kyle RA , Larson DR , Therneau TM , et al. Long‐term follow‐up of monoclonal gammopathy of undetermined significance. N Engl J Med. 2018;378:241‐249. 29342381 10.1056/NEJMoa1709974 PMC5852672

[9] Heilman RL , Velosa JA , Holley KE , Offord KP , Kyle RA . Long‐term follow‐up and response to chemotherapy in patients with light‐chain deposition disease. Am J Kidney Dis. 1992;20:34‐41. 1621676 10.1016/s0272-6386(12)80314-3

[10] Gabbay E , Shavit L , Ganzel C , et al. The daughter of time: late development of Waldenstrom’s Macroglobulinemia in a patient with immunotactoid glomerulopathy. J Hematol Oncol Res. 2015;2:29‐34.

[11] Sethi S , Rajkumar SV . Monoclonal gammopathy‐associated proliferative glomerulonephritis. Mayo Clin Proc. 2013;88:1284‐1293. 24182705 10.1016/j.mayocp.2013.08.002

[12] Gumber R , Cohen JB , Palmer MB , et al. A clone‐directed approach may improve diagnosis and treatment of proliferative glomerulonephritis with monoclonal immunoglobulin deposits. Kidney Int. 2018;94:199‐205. 29759418 10.1016/j.kint.2018.02.020

